# Understanding the role of *Staphylococcus aureus* in atopic dermatitis: strain diversity, microevolution, and prophage influences

**DOI:** 10.3389/fmed.2024.1480257

**Published:** 2024-11-18

**Authors:** Zhongjie Wang, Claudia Hülpüsch, Claudia Traidl-Hoffmann, Matthias Reiger, Michael Schloter

**Affiliations:** ^1^Research Unit for Comparative Microbiome Analysis, Helmholtz Munich, German Research Center for Environmental Health, Neuherberg, Germany; ^2^Department of Environmental Medicine, Faculty of Medicine, University of Augsburg, Augsburg, Germany; ^3^Insitute of Environmental Medicine, Helmholtz Munich, German Research Center for Environmental Health, Neuherberg, Germany; ^4^Christine Kühne Center for Allergy Research and Education, Davos, Switzerland; ^5^Chair of Environmental Microbiology, TUM School of Life Sciences Weihenstephan, Technical University of Munich, Freising, Germany

**Keywords:** atopic dermatitis, *Staphylococcus aureus*, strain diversity, microevolution, prophage, antibiotic resistance, horizontal gene tranfer

## Abstract

Atopic dermatitis (AD) is a prevalent inflammatory skin disorder characterized by chronic inflammation, skin barrier dysfunction, and microbial dysbiosis, with *Staphylococcus aureus* playing a significant role in its pathogenesis. This paper explores the strain diversity and microevolution of *S. aureus* within AD patients, emphasizing how specific strains adapt to the altered skin environment, exacerbating the condition. The review emphasizes the significance of variation in specific functional genes among *S. aureus* strains, which enhances their ability to adapt to different microenvironments and shapes their pathogenic potential. It also discusses how mobile genetic elements, particularly prophages, contribute to genetic diversity and drive the virulence and antibiotic resistance of *S. aureus* in AD, highlighting the clinical challenges posed by these strain-specific factors in managing the disease. The paper advocates for the integration of advanced genomic tools such as whole-genome sequencing and machine learning to develop targeted therapies. By focusing on the genetic adaptability of *S. aureus* and its impact on AD, this review underscores the need for strain-specific diagnostics and personalized treatment strategies to improve patient outcomes.

## 1 Introduction

*S. aureus* is a highly adaptable and versatile bacterium known for its ability to inhabit a wide range of hosts, including humans (1), livestock, and domestic pets (2, 3), suggesting a potential for cross-species transmission. It has also been detected in various environmental settings (4). This bacterium has a long evolutionary history as both a commensal and an opportunistic pathogen, reflecting its remarkable ability to thrive in diverse ecological niches. In humans, *S. aureus* commonly colonizes the nose, ear, and skin as a commensal bacterium in about 20–30% of the population (1). However, its presence is not purely neutral; *S. aureus* is also associated with several atopic diseases, such as AD (5) and hay fever in the nose (6), demonstrating its dual role in human health as both a harmless inhabitant and a potential trigger for allergic reactions.

The ability of *S. aureus* to exist in such a wide range of environments and hosts raises significant questions about its genetic adaptability: does the core genome of *S. aureus*, which includes genes common to all strains, provide the necessary flexibility for adaptation and phenotypic changes across different ecological niches, or is it possible that the pan-genome, which encompasses the entire set of genes present across various strains of *S. aureus*, allows specific strains to colonize distinct niches? For example, in the context of AD, the affected skin regions can be considered as a distinctive environment with an increased selection pressure, which might promote the evolution of *S. aureus* (7, 8). Therefore, understanding the drivers for microevolution of *S. aureus* will improve our understanding of the commensal or pathogenic nature of strains which will allow for a more precise identification of pathogenic strains, which might facilitate in the future the development of new forms of therapy for AD patients.

In this paper, we summarize the current stage of knowledge on strain diversification of *S. aureus* in AD patients and vote for a strain-specific identification of *S. aureus* also for diagnostic purposes.

## 2 Discussion

### 2.1 *Staphylococcus aureus* prevalence in AD patients

AD stands as a significant clinical and public health concern worldwide, characterized by chronic inflammation, intense itching, and a compromised skin barrier (9). The global prevalence of AD has been increasing over the past decades, affecting over 20% of children and 10% of adults worldwide (10). The 2019 Global Burden of Diseases data shows 171 million cases of AD worldwide, with a 28.6% increase in prevalence since 1990 (133 million) and a strong association with higher socioeconomic development (11). This rise not only highlights the pressing need for effective management strategies but also underscores the multifactorial nature of AD, influenced by genetic, immunological, psychological, and environmental factors like the skin microbiome (12).

Central to this discussion on AD pathology is the role of *S. aureus*. While *S. aureus* often coexists harmlessly with its host, its colonization is not just prevalent in individuals with AD but also plays a pivotal role in exacerbating the condition. Numerous studies have revealed a significantly higher rate of *S. aureus* colonization in AD patients compared to healthy individuals (13–15). The primary source of *S. aureus* contamination in AD patients is self-colonization from the nasal passages (11), spreading to the skin through direct contact, especially in areas with a compromised skin barrier. Research indicates that up to 70–90% of AD patients are colonized by *S. aureus* on lesional skin, a strong contrast to 20–30% in healthy individuals colonized by *S. aureus* in the nose (16, 17). This heightened prevalence is not limited to lesional skin; non-lesional skin of AD patients also exhibits higher colonization rates (17, 18). AD is more prevalent and severe in children, particularly infants and young children aged 5–9 years, while adults experience a lower prevalence and more localized symptoms as they age. In pediatric AD patients, the prevalence of *S. aureus* nasal colonization ranges from 46.4 to 82.2%, compared to 19.4 to 34.1% in the control group, while in adults with AD, nasal colonization rates are between 62.0 and 69.8% (19). For skin colonization, the rate of *S. aureus* increases with age in AD patients, with colonization rates of 50% in infants, 80% in children, and 87.5% in adults for acute lesions, and 18.5% in infants, 41.8% in children, and 48.9% in adults for chronic lesions (20). This increased burden of *S. aureus* correlates with both skin microbiome dysbiosis and heightened disease severity during flares (13, 14).

### 2.2 Pathogenic mechanisms of *Staphylococcus aureus* in AD patients

**Figure 1:**
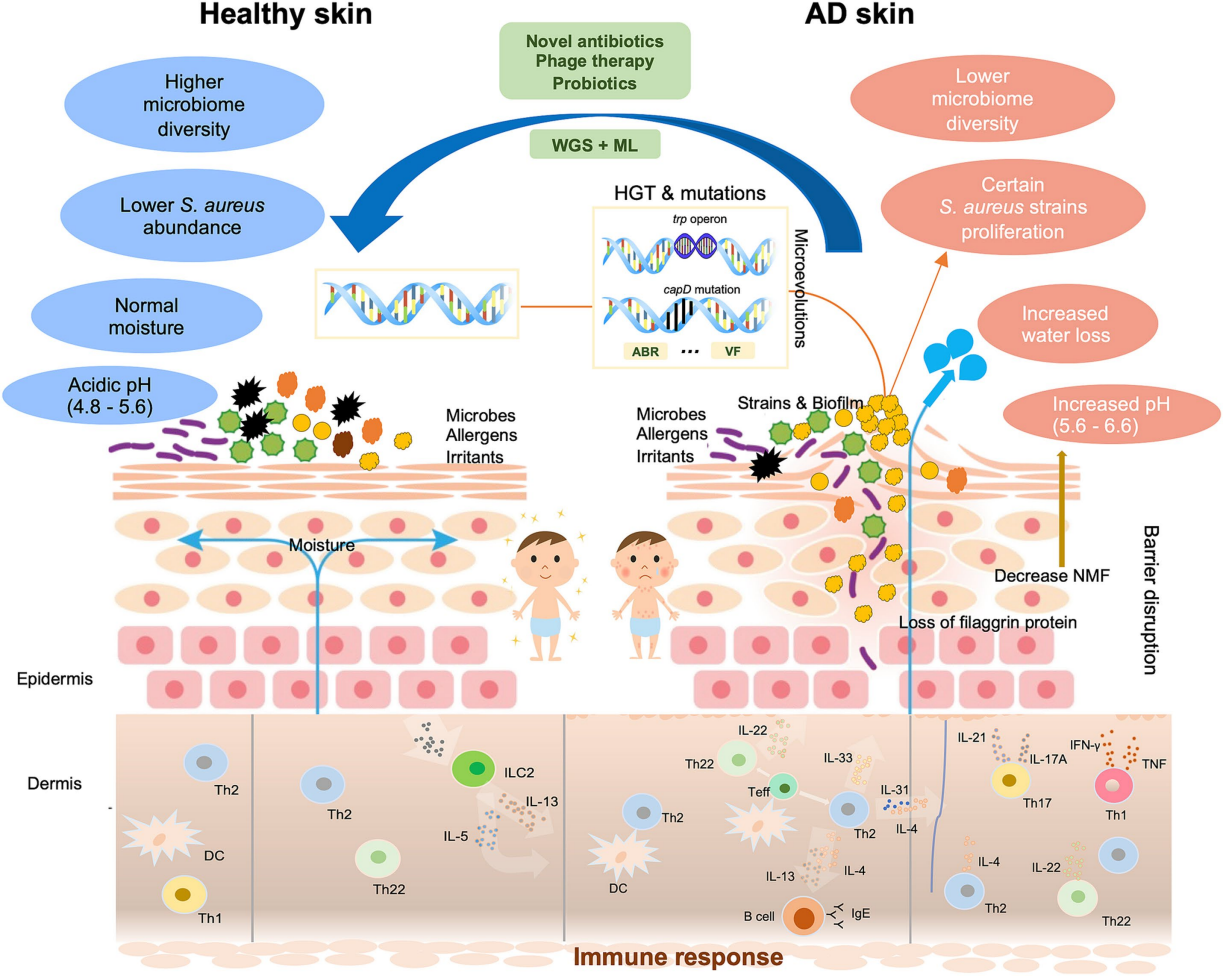
Schematic diagram of the multifaceted impact of microevolution on the pathogenicity of *Staphylococcus aureus* in AD. Normal skin is clinically intact with balanced diverse bacteria and physiological traits. AD skin exhibits the disrupted epidermal barrier like the flaggrin gene mutation, reduced skin microbiome diversity, increased water loss, and elevated pH. In affected skin areas where the altered microenvironment exerts significant selection pressure on certain *Staphylococcus aureus* strains, these strains proliferate, and heightened immune responses are triggered: Langerhans cells and inflammatory epidermal dendritic cells, which carry specific IgE attached to the high-affinity IgE receptor, as well as dermal dendritic cells, facilitate the introduction of allergens and antigens. Cytokines IL-4, IL-13, and IL-31 of the type-2 class directly stimulate sensory nerves, leading to itchiness. These changes make AD skin more susceptive to allergens, irritants, and pathogens. Mutation and HGT may occur in certain AD-associated strains, which result in increased production of VFs, and acquisition of ABR genes, and genetic variations that allow better adhesion (*capD* gene mutation), enhanced metabolic capabilities (*trp* operon), biofilm formation. Proposed therapeutic approaches include phage therapy, probiotics, and the integration of WGS and ML to develop targeted treatments. WGS, whole genome sequencing. ML, machine learning. ABR, antibiotic resistance. VF, virulence factor. HGT, horizontal gene transfer. DC, dendritic cell. IFN, interferon. IL, interleukin. ILC, innate lymphoid cell. Teff, effector T cell. Th, T-helper cell. TNF, tumor necrosis factor.

The impact of *S. aureus* colonization on AD symptoms is profound and multifaceted, involving the direct effects of bacterial colonization, the impact of toxins produced by *S. aureus*, and the induction of inflammatory responses that worsen AD symptoms (Figure 1). The abnormal proliferation of *S. aureus* in AD leads to dysbiosis of the skin microbiome, significantly reducing overall microbial diversity and weakening the skin barrier. This increases susceptibility to irritants, allergens, and pathogens (21), disrupts the skin immune response, and exacerbates inflammation (22). Recent studies reveal that early-onset barrier dysfunction triggers immune responses to commensal organisms, with *S. aureus* forming antimicrobial-resistant biofilms that influence keratinocyte biology and contribute to AD-associated inflammation (23). In addition, IL-9 is differentially produced by skin-tropic and extracutaneous memory T cells in response to various allergens like house dust mites and staphylococcal enterotoxin B, enabling patient stratification based on allergen sensitization and highlighting the key role of *S. aureus* in immune polarization in AD patients (24). Genetic mutations, such as those in the filaggrin gene, impair the skin barrier and create a favorable environment for *S. aureus* colonization (25). Psychological factors like stress impose unneglectable influences on the skin’s immune response, as stress-induced neuropeptide mediators affect immune and skin cells, increasing mast cell activity and nerve interactions (26, 27). Yet, direct studies specifically linking stress to particular strains of *S. aureus* are limited. The altered skin microenvironment in AD, including reduced antimicrobial peptide production (28) and increased skin pH, further supports *S. aureus* proliferation in a pH-dependent manner (29).

*S. aureus* binds more efficiently to AD skin due to specific receptors and binding proteins like fibronectin-and fibrinogen-binding proteins, which are upregulated in AD’s inflammatory milieu (30, 31). Once colonized, *S. aureus* exacerbates AD through the production of virulence factors (VF) that act as superantigens, inducing a disproportionate immune response and increased inflammation (30, 32). *S. aureus* also secretes enzymes like proteases and lipases that degrade crucial skin barrier proteins, increasing permeability and facilitating the entry of other allergens and pathogens (33, 34). Furthermore, the ability of *S. aureus* to form biofilms complicates eradication, as biofilms resist antimicrobial agents and host immune responses, making colonization persistent (35). These interactions highlight the need for targeted therapies addressing both the pathogen and immune dysregulation to effectively manage AD.

### 2.3 Strain diversity, microevolution, and geographical scale for *Staphylococcus aureus* diversification

The clinical implications of *S. aureus* in AD are significantly influenced by its strain diversity. Previous research has shown significant differences between *S. aureus* strains from AD patients and healthy individuals (14, 36, 37), indicating that AD-associated strains have a selective advantage in inflamed skin. Additionally, studies further revealed a high degree of strain diversity of *S. aureus* within AD (7, 8), with specific strains or clonal complexes (CC) of *S. aureus* like CC1 being more frequently associated with AD (36, 38), highlighting the significance of strain diversity.

The diversity of *S. aureus* strains complicates the management of AD in several ways.

1. Certain strains might be more pathogenic than others due to their secretion of strain-specific VFs. Understanding these strain-specific factors is crucial for developing targeted interventions. For instance, some strains exhibit heightened virulence through the production of specific toxins or enzymes that support cell wall anchoring and thus further disrupt the skin barrier or modulate the immune response more aggressively, leading to more severe AD flare-ups (25, 39). The variation in superantigens activates distinct subsets of T cell receptors, giving *S. aureus* strains unique T cell-avoidance capabilities, resulting in persistent infections and chronic inflammation (40).
2. *S. aureus* strains might possess distinct unique antibiotic resistance (ABR) profiles, which compounds the challenge of targeting *S. aureus,* especially for severe inflammatory AD conditions. In the UK, researchers found a higher prevalence of specific *S. aureus* CCs in AD patients, with over 80% of these strains carrying a plasmid with the ß-lactamase gene, conferring penicillin resistance—an attribute not found in the control group (36). Similarly, *S. aureus* isolates from AD showed higher fusidic acid resistance (41), making infections harder to treat.
3. *S. aureus* may possess strain-specific functions or pathways that confer a selective advantage in the AD microenvironment. A cohort study from Japan discovered that dysfunctional mutations in the accessory gene regulatory (*Agr*) quorum-sensing system were more common in the HE group, linking a functional *Agr* system to AD (42). An Japanese study discovered that only ST97_A_, a subclade of *S. aureus* detected in lesional AD skin, possessed the complete tryptophan (*trp*) operon, enabling bacterial survival without exogenous *trp* on AD skin, where the *trp* level was significantly reduced (43). A recent global study revealed that adaptive capsule loss via *capD* mutations, essential for *S. aureus* capsular polysaccharide synthesis, was more common in AD, enhancing adherence to AD skin (7). Studies also showed that *S. aureus* strains exhibit heterogeneous siderophore production within the host (44). In the context of AD, *S. aureus* strains with robust siderophore production can dominate the skin microbiome by outgrowing other bacteria with less efficient iron scavenging mechanisms. This competitive edge can lead to a higher relative abundance of *S. aureus* in AD patients, exacerbating the condition due to its pathogenic potential.

These results underscore the critical importance of strain-level variation in *S. aureus*, highlighting the need for precise and individualized approaches in managing AD. Furthermore, these findings emphasize the necessity of considering geographical scale when examining the diversification mechanisms of *S. aureus* strains. Most observed differences have been region-specific, suggesting these adaptations are spontaneous rather than indicative of a general distinction between AD and HE strains. The broader question of whether these genomic differences and adaptations occur locally or globally remains unresolved. Additionally, a recent study revealed that patient co-factors such as age, sex, and race also significantly impact the variation (5). This raises relevant questions: to what extent do health status, geographical location/scale, and patient co-factors influence *S. aureus* diversification? Which factors are more critical? Furthermore, given the frequent interactions between humans, livestock, and pets, could these animals serve as hotspots for microevolutionary changes? These questions underscore the complex interplay of factors affecting *S. aureus* diversification.

### 2.4 Role of prophages in *Staphylococcus aureus* adaptability and pathogenicity

Overall, around 15–20% of *S. aureus* genome is characterized by the presence of numerous mobile genetic elements such as plasmids, transposons, and bacteriophages (45). This genomic plasticity could be a key driver of its pathogenic potential. These MGEs facilitate genetic exchanges, conferring new metabolic capabilities and enabling the rapid acquisition of genes encoding ABRs and VFs. Such genomic adaptability allows *S. aureus* to swiftly respond to environmental selective pressures, making it a formidable pathogen.

Bacteriophages, known for their host specificity and diverse functional gene repertoire (46), significantly influence bacterial evolution. Embedded within the bacterial genome, prophages are bacteriophage DNA that can be lytic or lysogenic. In particular, lysogenic prophages, dormant within the host genome, could introduce new genes, enhance bacterial metabolic capabilities, and potentially increase pathogenicity, leading to important clinical implications (47). Research indicates that the primary mechanism of horizontal gene transfer in *S. aureus* is bacteriophage-mediated transduction (48). Therefore, prophages play a pivotal role in the diversification and pathogenicity of *S. aureus*, significantly influencing its genetic diversity, virulence, and ABR (57). For example, prophages in *S. aureus* sequence type 398 carry a set of genes encoding VFs, including a tyrosine recombinase linked to biofilm-associated staphylococcal infections, an ATP-dependent Clp protease, and an autolysin Atl involved in mediating adhesion to host tissue (49). Despite advances in understanding *S. aureus* genetic diversity, the extent to which prophages contribute to this genetic diversity and their link to AD severity remains underexplored. Therefore, profiling the prophages in *S. aureus* strains is crucial for understanding the relationship between genomic composition, phage-driven adaptation, and resultant phenotypic outcomes of *S. aureus* strains.

### 2.5 Future directions in research and treatment

AD is a prevalent and complex condition posing a significant global public health challenge. The diverse strains of *S. aureus* in AD, varying in virulence, ABR, and metabolic potential, call for advanced research methodologies and innovative treatment strategies.

Traditional treatments for AD, such as systemic glucocorticosteroids and ciclosporin, remain effective for more severe cases. In addition, therapies like topical or systemic antibiotics, topical corticosteroids, calcineurin inhibitors, and supportive options like bleach baths or antiseptics washes are often recommended. However, frequent use of antiseptics can disrupt the balance of the skin’s microbial community, thus should be limited to specific cases and combined with strategies aimed at restoring and maintaining a healthy skin microbiome. Meanwhile, new therapies have been introduced, including biologics like dupilumab and tralokinumab, along with Janus kinase inhibitors such as abrocitinib, baricitinib, and upadacitinib, which offer targeted immunomodulatory effects (50). Addressing psychological stress through behavioral therapy and psychopharmacologic agents (e.g., anxiolytics) could be an additional measure.

Whole-genome sequencing (WGS) and machine learning (ML) technologies offer unprecedented opportunities to decipher the subtle differences between *S. aureus* strains from AD and HE (51, 52). WGS has revolutionized our understanding of the genetic diversity of *S. aureus*, allowing for the detailed characterization of strain-specific markers and the identification of VF and ABR genes (53). When coupled with ML algorithms, WGS data can be analyzed to predict strain pathogenicity, ABR profiles, and potential VFs with high accuracy. This integrative approach facilitates the identification of novel biomarkers for *S. aureus* strains that are specifically associated with AD severity, enabling the development of more advanced diagnostic tools and personalized treatment approaches. Additionally, high-throughput isolation techniques are needed, as current molecular data may lack the necessary resolution for strain-level analyses. Moreover, creating and studying mutants can offer valuable insights into gene functions by allowing researchers to observe the effects of specific gene deletions or modifications, which can uncover potential targets for therapeutic intervention. To gain a systematic perspective, a global approach to characterizing strain variation and *S. aureus*’s role in AD across different populations and regions can provide valuable insights. Furthermore, alternative therapies like phage therapy and probiotics show promise in restoring skin microbiome balance (54–56) and reducing the risk of dysbiosis associated with broad-spectrum antibiotics. By employing these advanced methods, we can potentially uncover new therapeutic targets or diagnostic markers, facilitating personalized treatment approaches and the development of targeted therapies.

## 3 Conclusion

In conclusion, this review underscores the critical role of *S. aureus* strain diversity and microevolution in the pathogenesis of AD. The variation in specific functional genes among *S. aureus* strains enables these bacteria to adapt to the unique microenvironment of AD-affected skin, contributing to their increased pathogenic potential. Additionally, the presence of MGEs, especially prophages, further drives the genetic variability and virulence of these strains, complicating the clinical management of AD. The review highlights the necessity of incorporating advanced genomic tools, such as WGS and ML, to better understand the complex interactions between *S. aureus* and the host, ultimately leading to more precise diagnostics and personalized treatment strategies. Addressing these strain-specific factors is essential for developing effective therapies that target the root causes of *S. aureus* pathogenicity in AD, potentially improving patient outcomes and reducing disease burden.
